# Root-Associated Entomopathogenic Fungi Modulate Their Host Plant’s Photosystem II Photochemistry and Response to Herbivorous Insects

**DOI:** 10.3390/molecules27010207

**Published:** 2021-12-29

**Authors:** Julietta Moustaka, Nicolai Vitt Meyling, Thure Pavlo Hauser

**Affiliations:** Department of Plant and Environmental Sciences, University of Copenhagen, Thorvaldsensvej 40, 1871 Frederiksberg, Denmark; nvm@plen.ku.dk

**Keywords:** photosynthetic efficiency, compensatory process, chlorophyll fluorescence imaging, herbivory costs, non-photochemical quenching, singlet oxygen, *Solanum lycopersicum*, *Spodoptera exigua*, *Metarhizium brunneum*, *Beauveria bassiana*

## Abstract

The escalating food demand and loss to herbivores has led to increasing interest in using resistance-inducing microbes for pest control. Here, we evaluated whether root-inoculation with fungi that are otherwise known as entomopathogens improves tomato (*Solanum lycopersicum*) leaflets’ reaction to herbivory by *Spodoptera exigua* (beet armyworm) larvae using chlorophyll fluorescence imaging. Plants were inoculated with *Metarhizium brunneum* or *Beauveria bassiana*, and photosystem II reactions were evaluated before and after larval feeding. Before herbivory, the fraction of absorbed light energy used for photochemistry (Φ*_PSII_*) was lower in *M. brunneum*-inoculated than in control plants, but not in *B. bassiana*-inoculated plants. After herbivory, however, Φ*_PSII_* increased in the fungal-inoculated plants compared with that before herbivory, similar to the reaction of control plants. At the same time, the fraction of energy dissipated as heat (Φ*_NPQ_*) decreased in the inoculated plants, resulting in an increased fraction of nonregulated energy loss (Φ*_NO_*) in *M. brunneum*. This indicates an increased singlet oxygen (^1^O_2_) formation not detected in *B. bassiana*-inoculated plants, showing that the two entomopathogenic fungi differentially modulate the leaflets’ response to herbivory. Overall, our results show that *M. brunneum* inoculation had a negative effect on the photosynthetic efficiency before herbivory, while *B. bassiana* inoculation had no significant effect. However, *S. exigua* leaf biting activated the same compensatory PSII response mechanism in tomato plants of both fungal-inoculated treatments as in control plants.

## 1. Introduction

The escalating demand for food supply worldwide and the necessity of more sustainable agricultural practices [1,2] has led to an increasing interest in the application of beneficial microbes in agriculture [3,4]. Entomopathogenic fungi are identified by their ability to infect insects and produce spores that can grow, germinate, and spread from the cadaver [5,6]. Most literature on entomopathogenic fungi has focused on their use for biocontrol of multiple arthropod agricultural pests [7,8,9]. Recently, however, researchers have discovered that these fungi can also associate with many plant species as rhizosphere colonizers or endophytes, and that associations of plants with entomopathogenic fungi can promote their defense against phytopathogens [5,10,11,12,13] and insect pests [3,14,15,16,17,18,19], depending on the isolate and the plant species. Thus, several studies in the last decades have focused on the effect of entomopathogenic fungi on plants’ defense and other plant properties [3,14,15,16,17,18,19,20]. These symbiotic effects suggest that entomopathogenic fungi may be exceptional tools for Integrated Pest Management (IPM) strategies [6,10].

The precise mechanism of the defense promotion in this symbiotic relationship is not fully understood. It can involve induction or priming of systemic resistance and production of defense compounds, in addition to increased nutrient availability for the plant. Thus, it has been shown that five species of *Metarhizium* and the species *Beauveria bassiana* can transfer insect-derived nitrogen to their plant hosts [21,22]; in return, plants provide the entomopathogenic fungi with photosynthates [23]. 

The ability of a plant to maintain its photosynthetic efficiency, even when attacked by antagonists, is crucial for the plant’s defense and compensatory responses [24,25]. Moreover, non-photochemical quenching (NPQ) is considered as the principal photoprotective mechanism in plants that dissipates excess light energy as heat and protects light reaction under biotic and abiotic stress conditions, preventing the formation of reactive oxygen species (ROS) [26,27,28,29]. NPQ is involved in the mechanism of plant acclimation to biotic or abiotic stress and has been suggested to be a major component of the systemic acquired resistance [30,31,32]. Associations of plants with another group of root-associated fungi, mycorrhizal fungi, are known to increase the photosynthetic efficiency of plants [33,34,35,36,37,38]; several studies have addressed how this is affected by environmental stress [35,37,38], including herbivore attack [39]. The effects of root-associated entomopathogenic fungi on plant’s photosynthesis are far less studied. When durum wheat (*Triticum durum* L. cv. Calero) was inoculated with *Metarhiziumbrunneum*, net photosynthesis and chlorophyll concentrations increased [40,41]. Inoculations of date palms (*Phoenix dactylifera* L.) with *B. bassiana* upregulated genes encoding photosynthesis-related proteins [42]. Furthermore, inoculation of potato (*Solanum tuberosum*) with encapsulated *M. brunneum* increased the maximum (F*v*/F*m*) and the effective quantum yield (Φ*_PSII_*) of photosystem II in poor nutrient conditions but not in fertilized soil [43]. However, to our knowledge no studies have shown that the root association with entomopathogenic fungi affects the plant photosynthetic response to insect herbivory.

In a previous study [44], we showed that photosynthetic efficiency of tomato leaves increased in response to biting by *Spodoptera exigua* larvae (Hűbner; beet armyworm) in neighboring leaf parts, suggesting a compensatory response. Tomato, *Solanum lycopersicum*, is one of the commonly cultivated horticultural crops [45,46] that is susceptible to a variety of insect pests [3,47,48]. One of these is *S. exigua*, a polyphagous species that consumes a wide variety of plant hosts, including many crops, causing severe economic losses [49,50,51].

In the present study, we investigate whether root-inoculations with two different entomopathogenic fungi, *Metarhizium brunneum* and *Beauveria bassiana*, increase the photosynthetic efficiency of tomato leaves, and whether the associations improve the leaf’s photosynthetic response to short-term herbivory by *S. exigua* larvae. For this, we used chlorophyll fluorescence imaging analysis, which allowed us to distinguish the localized leaf response at the feeding spots and in the other leaflet areas.

## 2. Results

### 2.1. Comparison of Untreated Control Plants with Treated (Triton X) Control Plants 

We used two different control treatments, an untreated control where the inoculum only contained tap water and a treated control inoculated with Triton X 0.05%. No significant difference was detected between the two controls for quantum yield of PSII photochemistry (Φ*_PSII_*), quantum yield of regulated non-photochemical energy loss in PSII (Φ*_NPQ_*), non-photochemical quenching (NPQ), electron transport rate (ETR), and excess excitation energy (EXC). For these parameters, values for the two control treatments were merged in subsequent analyses. The two control treatments did differ significantly for quantum yield of nonregulated energy loss in PSII (Φ*_NO_*) and photochemical quenching (q_P_) before herbivory, where Φ*_NO_* and q_P_ were 8% and 15% lower in the treated control, respectively (Table S1). These values were not merged for the two control treatments in the analyses.

### 2.2. Effect of Root-Associated Entomopathogenic Fungi on Light Energy Distribution of Photosystem II before and after Herbivory

#### 2.2.1. Before Herbivory

The absorbed light energy in PSII is used either for photochemistry (Φ*_PSII_*), is photoprotectively dissipated as heat (Φ*_NPQ_*), or is nonregulated loss (Φ*_NO_*). The sum of these three fractions adds to unity [52].

**Figure 1:**
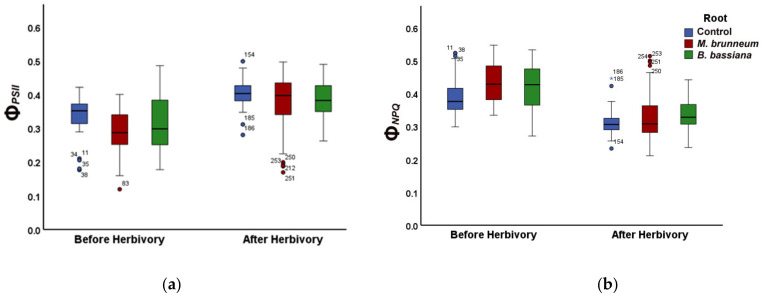
Changes in PSII quantum yields, (**a**) effective quantum yield of photochemistry (ΦPSΙΙ), and (**b**) regulated non-photochemical energy loss (ΦNPQ) in tomato leaflets before and immediately after herbivory 15 min of insect feeding, shown for untreated and treated controls (combined, blue), M. brunneum-inoculated plants (red), and B. bassiana-inoculated plants (green). Boxes and whiskers indicate the minimum, first quartile, median, third quartile and maximum, circles and asterisks indicate outliers, for statistical significance, see Supplementary Table S2.

**Figure 2:**
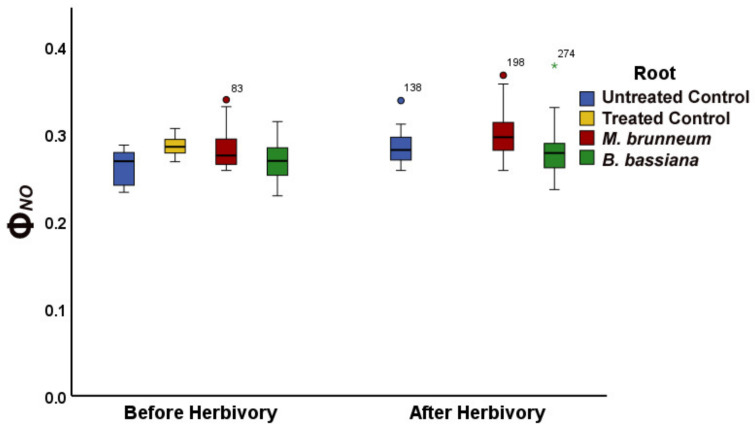
Nonregulated energy loss (ΦNO) of tomato leaflets before and immediately after insect feeding, from before to after herbivory, shown for untreated and treated controls (blue and yellow; or combined in blue). M. brunneum-inoculated plants (red); B. bassiana-inoculated plants (green). Boxes and whiskers explained in Figure 1. For statistical significances, see Supplementary Table S3.

In *M. brunneum*-inoculated tomato plants, the fraction of energy used for photochemistry (Φ*_PSII_*) decreased by 15% compared with that of control plants (Figure 1a; Table S2). At the same time, the fraction energy dissipated as heat (Φ*_NPQ_*) increased by 12% compared with control (Figure 1b; Table S2), consequently, having an increase of 8% of the fraction of energy that is lost nonregulated in PSII (Φ*_NO_*), compared with the untreated (Figure 2; Table S3). However, there was no difference in Φ*_NO_* compared with the treated control (Figure 2; Table S3). 

In *B. bassiana*-inoculated plants, the fraction of absorbed light energy used for photochemistry (Φ*_PSII_*) did not differ from controls, whereas the regulated energy loss (Φ*_NPQ_*) was 9% higher and the nonregulated loss (Φ*_NO_*) was 6% lower than the treated control plants (Figure 1a,b and Figure 2; Tables S2 and S3) but had no difference compared with untreated control plants (Figure 2; Table S3).

#### 2.2.2. After Herbivory

**Figure 3:**
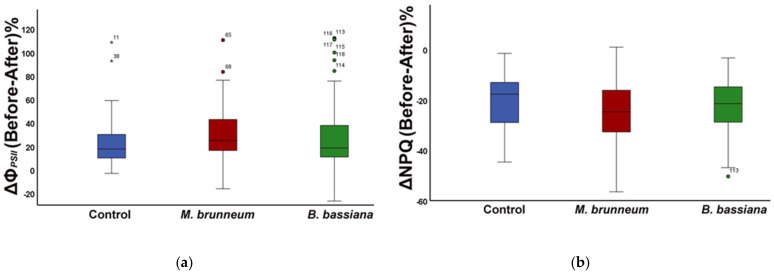
Difference of Before-After herbivory, expressed as percentage change, in the fraction of absorbed light energy (**a**) used for photochemistry (Φ*_PSΙΙ_*) and (**b**) dissipated as heat (Φ*_NPQ_*), shown for control plants (blue, untreated and treated combined), *M. brunneum*-inoculated (red), and *B. bassiana*-inoculated plants (green). Boxes and whiskers explained in Figure 1.

After herbivory, the fraction of absorbed light energy used for photochemistry (Φ*_PSII_*) did not differ between treatments (Figure 1a and Figure 3a; Table S2). In *B. bassiana*-inoculated plants, the regulated and nonregulated energy loss (Φ*_NPQ_* and Φ*_NO_*) was 7% higher and 2% lower than in control plants, respectively. Φ*_NPQ_* and Φ*_NO_* were 3% higher and 6% lower in *B. bassiana*-inoculated than in *M. brunneum*-inoculated plants, respectively (Figure 1b and Figure 3b; Table S2).

**Figure 4:**
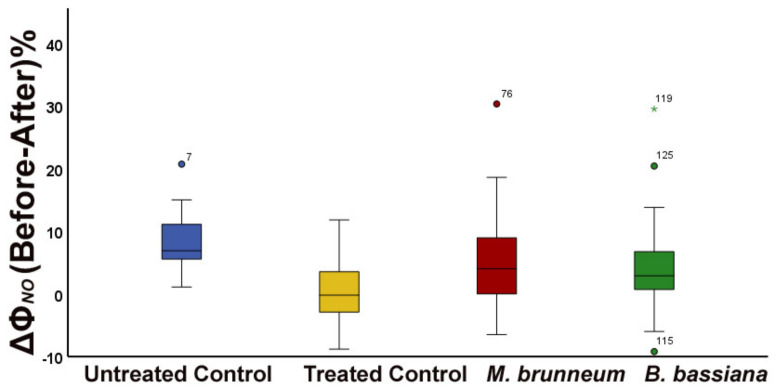
Difference of Before-After herbivory in the fraction of absorbed light energy lost nonregulated (Φ*_NO_*), expressed as percentage change, shown for untreated and treated control plants (blue and yellow), *M. brunneum*-inoculated (red), and *B. bassiana*-inoculated plants (green). Boxes and whiskers explained in Figure 1.

In *M. brunneum*-inoculated plants, the fraction of absorbed light energy used for photochemistry (Φ*_PSII_*) increased by 32% compared with that before herbivory (Figure 1a and Figure 3a; Table S2), while the fraction dissipated as heat (Φ*_NPQ_*) decreased 22% and the fraction of nonregulated heat loss (Φ*_NO_*) increased 5% (Figure 1b, Figure 2, Figure 3b, and Figure 4; Tables S2 and S3).

In *B. bassiana*-inoculated plants, Φ*_PSII_* increased compared with before herbivory by 24% (Figure 1a and Figure 3a; Table S2). Simultaneously, Φ*_NPQ_* decreased by 19% (Figure 1b and Figure 3b; Table S2) but without any difference in the amount of non-regulated energy loss (Φ*_NO_*) compared with before herbivory (Figure 2 and Figure 4; Table S3).

### 2.3. Effect of Root-Associated Entomopathogenic Fungi on the other Chlorophyll Fluorescence Parameters before and after Herbivory

#### 2.3.1. Before Herbivory

**Figure 5:**
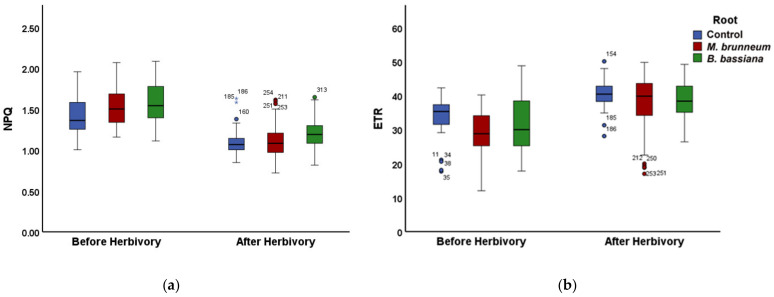
(**a**) Non-photochemical quenching (NPQ) and (**b**) electron transport rate (ETR) of tomato leaflets before and after insect feeding, shown for control plants (blue, untreated and treated combined) *M. brunneum*-inoculated (red), and *B. bassiana*-inoculated plants (green). Boxes and whiskers explained in Figure 1. For statistical significances, see Supplementary Table S4.

**Figure 6:**
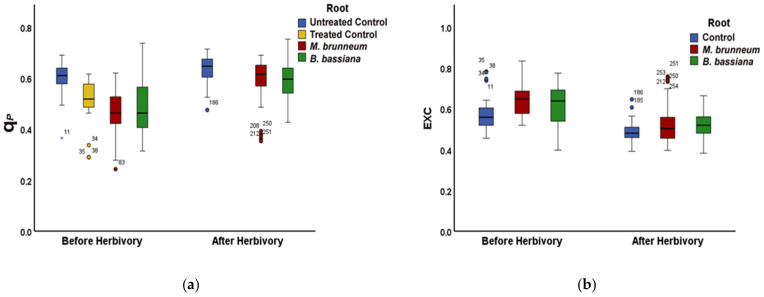
(**a**) Fraction of open PSII reaction centers (q*_p_*) and (**b**) excess excitation energy (EXC) of tomato leaflets before and after insect feeding, shown for untreated and treated control plants (blue, yellow; or combined), *M. brunneum*-inoculated (red), and *B. bassiana*-inoculated plants (green). Boxes and whiskers explained in Figure 1. For statistical significances, see Supplementary Tables S3 (q*_p_*) and S5 (EXC).

In *M. brunneum*-inoculated plants, the non-photochemical quenching (NPQ) was 9% higher than in control plants, resulting in a 15% decreased electron transport rate (ETR), possibly due to the decreased (22%) fraction of open reaction centers (q_P_) (Figure 5a,b and Figure 6a; Tables S3 and S4). At the same time, the excess excitation energy (EXC) increased by 12% compared with that of control plants (Figure 6b; Table S5).

In *B. bassiana*-inoculated plants, the non-photochemical energy quenching (NPQ) was also higher than in control plants (11%), which had no effect on the electron transport rate (ETR) (Figure 5a,b; Table S4). The fraction of open reaction centers (q*_P_*) was 18% lower than in control plants; in accordance, the excess excitation energy (EXC) was 8% higher than in control plants (Figure 6a,b; Tables S3 and S5).

#### 2.3.2. After Herbivory

In *B. bassiana*-inoculated plants, the non-photochemical energy quenching (NPQ) was 9% higher than in control plants and 9% higher than in *M. brunneum*-inoculated plants (Figure 5a and Figure S1a; Table S4). The control, *M. brunneum* treatment, and *B. bassiana* treatment did not differ for electron transport rate (ETR) or excess excitation energy (EXC) after herbivory (Figure 5b, Figure 6b, Figure S1b and S2b; Tables S4 and S5), but both *M. brunneum*- and *B. bassiana*-inoculated plants had 7% less open reaction centers than control plants (Figure 6a and Figure S2a; Table S3).

Plants inoculated with *M. brunneum* had a 22% and 19% lower NPQ and EXC after herbivory than before (Figure 5a, Figure 6b, Figure S1a and S2b; Tables S4 and S5), respectively. Simultaneously, they upregulated their electron transport rate by 32% and had 27% more open reaction centers than before herbivory (Figure 5b, Figure 6a, Figures S1b and S2a; Tables S4 and S3).

*B. bassiana*-inoculated plants had 23% and 16% lower NPQ and EXC than before herbivory, respectively (Figure 5a, Figure 6b, Figure S1a and S2b; Tables S4 and S5); similar to the *M. brunneum*-inoculated plants, they increased ETR (Figure 5b and Figure S1b; Table S4) and q*_P_* (Figure 6a and Figure S2a; Table S3) by 30% and 22% than before herbivory.

### 2.4. Spatial Heterogeneity of Photosystem II Photochemistry in Response to Herbivory by Spodoptera exigua larvae

**Figure 7:**
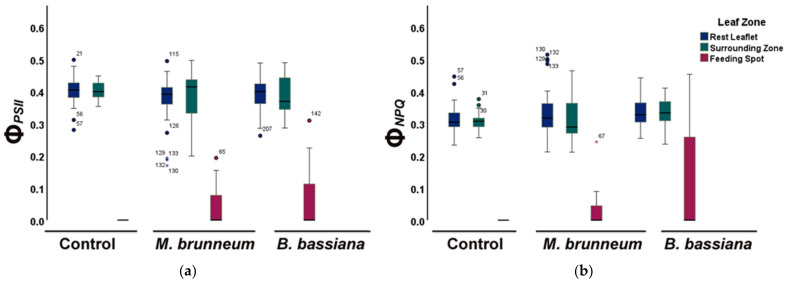
Fraction of absorbed light energy (**a**) used for photochemistry (Φ*_PSΙΙ_*) and (**b**) lost in regulated non-photochemical processes (Φ*_NPQ_*), shown for three different leaflet zones: directly at feeding spots (red), in zones surrounding the feeding spot (green), and in the rest of the leaflet (blue), for control, *M. brunneum*-inoculated, and *B. bassiana*-inoculated plants. Boxes and whiskers explained in Figure 1. For statistical significances, see Supplementary Table S6.

**Figure 8:**
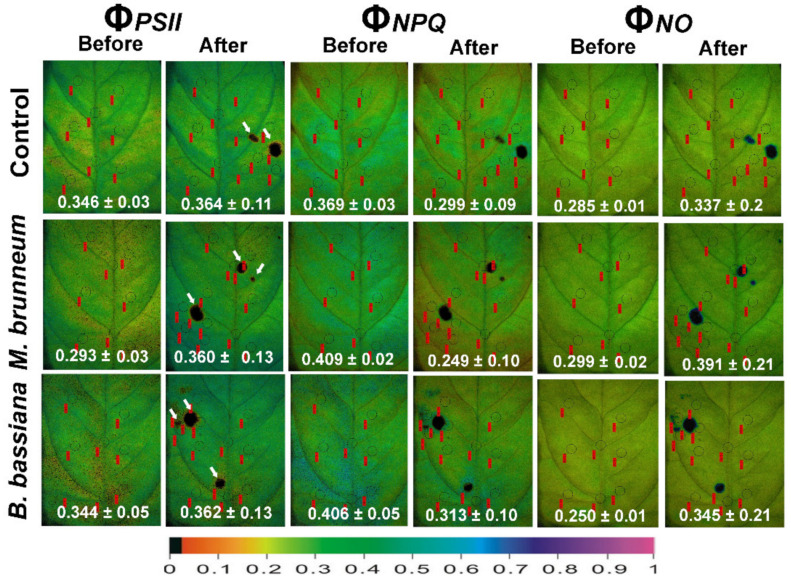
Representative color-coded images of the fraction of absorbed light energy, used for photochemistry (Φ*_PSΙΙ_*), dissipated as heat-regulated loss (Φ*_NPQ_*), and nonregulated loss (Φ*_NO_*) of tomato leaflets before and after insect feeding, for treated control plants, *M. brunneum*-inoculated, and *B. bassiana*-inoculated plants. Initial measurement areas (areas of interests, AOIs) are shown in circles with associated chlorophyll fluorescence values in red labels. After insect feeding, new AOIs were added to cover the feeding spots of herbivory (shown by white arrows). The whole leaflet’s corresponding values (average ± SD) are given in white. The color code at the bottom of the images ranges from pixel values 0.0 (black) to 1.0 (purple).

In both *M. brunneum*- and *B. bassiana*-inoculated plants, the fraction of energy used for photochemistry (Φ*_PSII_*) was higher in leaf-zones surrounding the feeding spots and the rest of the leaflets than at the feeding spots (Figure 7a and Figure 8; Table S6) similar to the control plants. However, Φ*_PSII_* of the rest of the leaflet of *M. brunneum*-inoculated plants was lower than the rest of the leaflet of control plants (Figure 7a and Figure 8; Table S6). The feeding spots of *B. bassiana*-inoculated plants had significantly higher Φ*_PSII_* compared with the feeding spot of control plants.

Both *M. brunneum*- and *B. bassiana*-inoculated plants had higher fractions of regulated energy dissipated as heat (Φ*_NPQ_*) in the zones surrounding the feeding spot as well as in the rest of the leaflet (Figure 7b and Figure 8; Table S6), same as the control plants. However, Φ*_NPQ_* at the feeding spots of *B. bassiana*-inoculated plants was significantly higher than on *M. brunneum*-inoculated and control plants (Figure 7b and Figure 8; Table S6).

**Figure 9:**
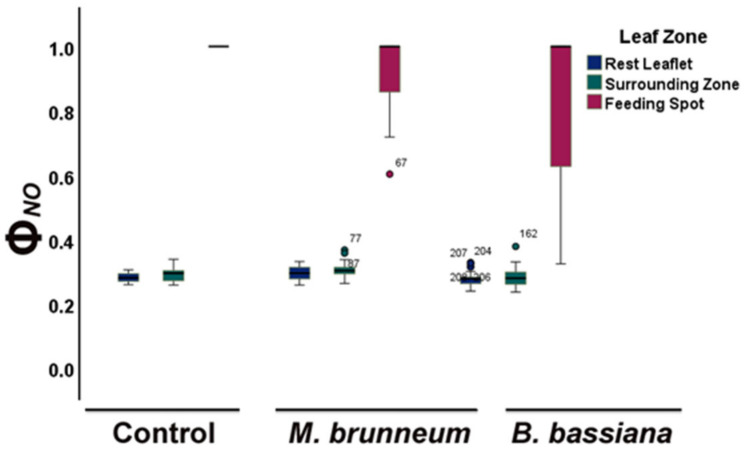
Fraction of absorbed light energy loss nonregulated (Φ*_NO_*), shown for three different zones: directly at feeding spots (red), in zones surrounding the feeding spots (green), and in the rest of the leaflet (blue), for control, *M. brunneum*-inoculated, and *B. bassiana*-inoculated plants. Boxes and whiskers explained in Figure 1. For statistical significance, see Supplementary Table S7.

Both *M. brunneum*- and *B. bassiana*-inoculated plants had lower non-regulated energy losses (Φ*_NO_*) in the zones surrounding feeding spots and the rest of the leaflet than at the feeding spots (Figure 8 and Figure 9; Table S7). Φ*_NO_* was significantly lower at the feeding spots of both *M. brunneum-* and *B. bassiana*-inoculated plants than at the feeding spots of control plants (Figure 8 and Figure 9; Table S7). In addition, Φ*_NO_* at the feeding spots was significantly lower in *B. bassiana*- than *M. brunneum*-inoculated plants (Figure 8 and Figure 9; Table S7).

## 3. Discussion

Plant colonization by beneficial fungi can stimulate the plant’s immune system, rendering the plant more resistant to herbivory by inducing systemic resistance, but the effect of beneficial fungi on plant relations with herbivorous insects is controversial [53,54,55,56,57], i.e., an efficient activation of the plant defense response upon pathogen or herbivory attacks [53]. For example, the leaf-chewer *Spodoptera exigua* had a higher mortality when feeding on tomato plants colonized by the mycorrhizal fungus *Funneliformis mosseae*, demonstrating mycorrhiza-induced resistance by accumulation of defense compounds [53]. 

However, the effects of associated fungi on plant relations with herbivorous insects are contradictory [54,55,58] with contrasting results, e.g., the effects of mycorrhiza on aboveground attackers. This suggests that “beneficial” root-associated fungi can either increase, decrease, or have no effect on plant tolerance-associated mechanisms [58]. Contradicting results have also been reported on the effect of inoculation with *B. bassiana* and *M. brunneum*, two fungi that are otherwise known as entomopathogens but can also associate with plant roots. *B. bassiana*-inoculation has been shown to reduce consumption of soybean leaves by *Helicoverpa gelotopoeon* larvae [59] but increase fecundity of second-generation aphids (*Aphis fabae)* on *Vicia faba* plants [60]. Inoculation with *M. brunneum* on soybeans increased the populations of the aphid *Aphis glycines* while *B. bassiana* inoculations had no effect [61].

Plant–fungus associations may also affect photosynthesis, which is highly important both for plant defense against antagonists and for compensatory responses. Inoculation of *Salvia fruticose* with the mycorrhizal fungus *Rhizophagus irregularis* increased the fraction of light energy used for photosynthesis (Φ*_PSII_*) and decreased the excess excitation energy (EXC) [33]. The effects of root-associated entomopathogenic fungi on the plant’s photosynthesis have not been studied before, to our knowledge, but may help in understanding and solving the contradictory effects of these fungi on plant interactions with antagonists.

In our study, the fraction of light energy used for photochemistry (Φ*_PSII_*) before herbivory was lower in *M. brunneum*-inoculated plants than in control plants, while there was no difference to controls in *B. bassiana*-inoculated plants (Figure 1a; Table S2), contradicting the effect of mycorrhizal fungi on Φ*_PSII_*. In both *M. brunneum*- and *B. bassiana*-inoculated plants, a higher fraction of energy was photoprotectively dissipated as heat (Φ*_NPQ_*) than in control plants (Figure 1b; Table S2). However, this photoprotective mechanism was sufficient only in *B. bassiana*-inoculated plants and not in *M. brunneum*, in which an increase of the quantum yield of nonregulated loss in PSII (Φ_NO_) was observed (Figure 2; Table S3). Φ*_NO_* comprises chlorophyll fluorescence internal conversions and intersystem crossing, resulting to singlet oxygen (^1^O_2_) formation through the triplet state of chlorophyll (^3^chl*) [27,33,62,63]. The ^1^O_2_ generated this way is a damaging reactive oxygen species (ROS) produced in PSII [64,65,66,67]. We can thus conclude that inoculation of tomato plants with *M. brunneum* may have a (slightly) negative impact on host plants under normal conditions, and that different entomopathogenic fungi differentially modulate PSII photochemistry. This is in accordance with literature reports, where different entomopathogenic fungi have different effects on plant and insect performance [68]. Even different *Metarhizium* species can affect photosynthesis differently [41,69,70].

After a short-term herbivory by chewing *S. exigua* larvae, the fraction of absorbed light energy used for photochemistry (Φ*_PSII_*) increased in both *M. brunneum*- and *B. bassiana*-inoculated plants as well as in control plants (Figure 1a and Figure 3a; Table S2). Simultaneously, the photoprotective heat dissipation (Φ*_NPQ_*) decreased in all treatments (Figure 1b and Figure 3b; Table S2) compared with before herbivory. These decreases in Φ*_NPQ_* resulted an increase in the nonregulated energy loss (Φ*_NO_*) in *M. brunneum*- but not in *B. bassiana*-inoculated plants (Figure 2 and Figure 4; Table S3). This was due to a higher non-photochemical quenching in *B. bassiana*-inoculated plants compared with control. Non-photochemical quenching (NPQ) can dissipate the excess light energy as heat, acting as a photoprotective mechanism of photosynthesis [71,72,73], regulating the energy that might lead to the creation of singlet oxygen (^1^O_2_). *Spodoptera exigua* larvae feeding on both *M. brunneum*- and *B. bassiana*-inoculated plants activated a compensatory PSII response mechanism that, by downregulating the systemic signaling of NPQ, upregulated their electron transport rate (ETR) (Figure 5a,b; Table S4). Defense response mechanisms can be triggered by NPQ so that light energy allocation is adjusted in order to have an enhanced PSII functionality [31]. Together, this suggests that tomato leaflets initiate a compensatory mechanism that increases their photosynthetic efficiency in response to herbivore feeding, but this is not substantially different between inoculated and noninoculated plants. In addition, despite the initial negative effect on photosynthesis by *M. brunneum*, the plants manage to activate the same compensatory mechanism as control plants to reach the same photosynthetic efficiency (Φ*_PSII_*) as control plants. In addition, *B. bassiana*-inoculated plants had higher photoprotection (higher NPQ, Figure 5a; Table S4) and lower formation of singlet oxygen (^1^O_2_).

In our spatial analyses, in both fungal treatments and control plants, the leaf zone surrounding the larval feeding spots and the remaining leaflet area had higher Φ*_PSII_* than before herbivory but also compared with the feeding spot. Both fungal treatments had higher Φ*_NPQ_* in their surrounding zone and the rest of the leaflet than the feeding spot. However, the Φ*_NPQ_* in the feeding spot of *B. bassiana*-inoculated plants was significantly higher compared with the *M. brunneum*-inoculated and control plants. Lastly, the feeding spot, as expected, had the highest Φ*_NO_* compared with the rest of the leaflet and the surrounding zone in both treatments. Nevertheless, the Φ*_NO_* of the feeding spot of *B. bassiana*-inoculated plants was significantly lower than that of *M. brunneum*-inoculated plants as well as control plants. During our experiments, we also observed that the individual *S. exigua* larvae tended to feed on more leaf spots within the tested leaflet of *B. bassiana*-inoculated plants but caused lower damage on each leaf spot (J. Moustaka pers. obs). This may explain the differences in the feeding spots of *B. bassiana*-inoculated plants in their Φ*_NPQ_* and Φ*_NO_*. Inoculation with *B. bassiana* has previously been found to enhance the levels of terpenoids on tomato plants, which resulted in reduced weight gain in *S. exigua* larvae feeding on the plants [50].

The compensatory reaction to herbivory can minimize a reduction in growth or reproduction of the plant after herbivore attack, improving fitness of plants growing in environments with a high herbivory level [74]. The compensatory capability differs, varying with the plant species, the extent of the leaf area lost, the timing of the herbivory, the environmental conditions, and the mode of herbivore injury [44,74]. Such a compensatory is rationalized by a higher requirement of the remaining leaf area for a higher fraction of the absorbed light energy for photochemistry to fix larger amounts of carbon [44,74]. The light reactions of photosynthesis feed the energy supply required for the production of compounds used in defense, such as hormones, and other defense-related metabolites [26,75].

Overall, our results suggest that before herbivory, *M. brunneum* had a negative effect on the photosynthetic efficiency of the tomato plants, whereas *B. bassiana* did not. After herbivory, both the inoculated and noninoculated plants upregulated their photosynthetic efficiency, both at the whole leaflet level but also locally in the zone surrounding the feeding spot. The ability of *M. brunneum*-inoculated plants to increase their photosynthetic efficiency after herbivory to the same level as control plants, despite their initial (before herbivory) negative effect, suggests a higher defense response. Associations with both entomopathogenic fungi, and especially *B. bassiana*, seemed to diminish the negative effects of herbivory on photosynthetic efficiency locally in the feeding spots. This suggests that *B. bassiana*-inoculated plant had an improved response to herbivory since these plants increased their photosynthetic efficiency, showing the activation of the same compensatory mechanism as noninoculated plants, but they also managed to regulate their excess energy by increasing their Φ*_NPQ_* instead of increasing the harmful nonregulated energy loss, Φ*_NO_*. We can conclude that inoculation of tomato plants with different entomopathogenic fungi differentially modulates PSII photochemistry.

## 4. Materials and Methods

### 4.1. Plant Material and Growth Conditions

Surface sterilized seeds (1 min in 70% ethanol followed by 10 min in 1% sodium hypochlorite, followed by 6 washes with sterilized water) of tomato plants (*Solanum lycopersicum* cv. Moneymaker; Kings seeds, Essex UK) were sown in 2 lt pots with potting soil (clay and silica; SW Horto AB, Hammenhög, Sweden). Plants were grown in a greenhouse for 5 weeks at 19 ± 1/17 ± 1 °C day/night temperature, with a photoperiod of 16-h day at 180 ± 20 μmol photons m^−2^ s^−1^ light, and 60 ± 5% relative humidity. Six-week-old plants were used for the experiments.

### 4.2. Spodoptera exigua

*Spodoptera exigua* larvae were cultured from eggs (Entocare Wageningen, Netherlands) on an artificial diet [44] prior to experiments and kept under controlled conditions at 21 ± 1 °C day/night temperature, with a 12-h light cycle and 38 ± 5% relative humidity. The larvae used in the experiments were L2 instar larvae, which were starved for 24 h before exposing them to the tomato leaflets, assuring a faster consumption.

### 4.3. Fungal Isolates and Suspensions

Two different root-associated entomopathogenic fungi were obtained from the collection of University of Copenhagen: *Metarhizium brunneum* KVL 16–36, which is isolated from the commercial product Met52 ^®^; *Beauveria bassiana* KVL 13–39, which is isolated from the commercial product BotaniGard ^®^. We chose to work with *B. bassiana* as it has already been broadly studied as a potential plant-associated entomopathogenic fungi with the ability to affect feeding of herbivorous insects and mites [3,55,68,76,77]. The species *M. brunneum* is less characterized for its effects on herbivory, but recently, has been shown to induce resistance against pests and pathogens [78]. Furthermore, isolate KVL 16–36 has been shown to alter the plant phenotype [79]. Both fungal isolates are obtained from commercial biocontrol products, which make them available for use in plant production systems in several countries. The fungal cultures were propagated in Saboraud Dextrose Agar plates (SDA) at 23 °C and darkness for 14–20 days. Fungal suspensions were prepared by adding 10 mL of Triton X 0.05% to the surface of the plates and scraping spores off with sterile glass spatula. The suspensions were filtered through sterile cheesecloth to remove the hyphal fragments and agar bits, centrifuged for 3 min at 3000× *g* rpm, and the supernatant was discarded. The precipitated spores were resuspended in 10 mL of Triton X 0.05%. The spore concentration was estimated by counting in a Fuchs–Rosenthal hemocytometer (Assistant, Sondheim von der Rhön, Germany, 0.0625 mm^2^, depth 0.200 mm) and adjusted to a final concentration of 1 × 10^8^ spores mL^−1^. The spore germination of the suspensions was tested by spreading a diluted sample (100 μL of 1 × 10^4^) on two SDA plates, after 24 h culturing, counting germinated and nongerminated spores. Suspensions with a germination rate of at least 90% were used for the experiment.

### 4.4. Experimental Design

Our experiment included four experimental plant treatments: untreated control, treated control, *Beauveria bassiana* inoculation, and *Metarhizium brunneum* inoculation, each with 6 replicates. On the sowing day, 1 ml of either tap water (Untreated Control), Triton X 0.05% (Treated Control), 1 × 10^8^ mL^−1^ spores of *Beauveria bassiana*, or *Metarhizium brunneum* was added to the surrounding of each seed. In each of the experimental plants, the terminal leaflet of the 4th leaf was used for the experimental measurements, as previously described [44]. Photosynthetic efficiency was measured before herbivory (Before) and immediately after a 15-min short feeding period by *S. exigua* (After). Subsequently, the roots of the plants were cleaned with tap water and a subsample containing primary and lateral roots was collected for assessment of fungal colonization, using a protocol modified from Steinwender et al. (2015) [80]. Briefly, roots were cut in 1-cm pieces and mixed to homogenize, after 20 pieces were selected randomly, added to a 15-mL glass tube containing 5 mL of sterile Triton X (0.05%) and homogenized with a rotating pestle. A 100 μL suspension was spread in selective media containing Streptomycin 0.5 mL of 0.6 g/mL, Tetracycline 0.5 mL of 0.05 g/mL, Cycloheximide 1 mL of 0.05 g/mL, and dodine 0.2 mL of 0.1 g/mL and incubated at 23 °C in darkness for 2 weeks. Colony Forming Units (CFU) that had the morphological units of the inoculated isolates were quantified on the 14th day for each plant (data not shown).

### 4.5. Chlorophyll Fluorescence Imaging Analysis

Chlorophyll *a* fluorescence was measured at room temperature using an imaging-PAM fluorometer (Walz, Effeltrich, Germany), as described previously [44]. Each leaflet was adapted in darkness for 15 min prior to measurement ensuring that all reaction centers were opened. Ten Areas of Interest (AOI) were added in each leaflet before herbivory and new AOIs were added after herbivory with one AOI in each feeding spot and two AOIs in the surrounding area of each feeding spot. Whenever a feeding spot was close to or inside an existing AOI (from the before measurement), it was considered as being in the surrounding AOI or the feeding spot in our analyses. For each AOI, we firstly measured and determined the minimum and the maximum chlorophyll *a* fluorescence in the dark (F*o* and F*m,* respectively). Steady-state photosynthesis (F*s*) was measured after 5 min of illumination time with Actinic Light (AL) of 200 μmol photons m^−2^ s^−1^ in accordance with the growth light conditions of the plants. Maximum chlorophyll *a* fluorescence in the light (F*m*‘) was measured with saturating pulses (SPs) every 20 s for 5 min after application of the AL. The minimum chlorophyll *a* fluorescence in the light (F*o*‘) was computed as F*o*‘ = F*o*/(F*v*/F*m* + F*o*/F*m*‘) [81] by the Imaging Win software. The variable chlorophyll *a* fluorescence (F*v*) in the dark-adapted leaves was calculated as F*m*-F*o*. The measured chlorophyll fluorescence parameters are shown in Table 1. Values were estimated as averages from six separate measurements.

The spatiotemporal response of the leaflets is displayed with representative color-coded images in Figure 8 obtained with 200 μmol photons m^−2^ s^−1^ AL.

### 4.6. Statistical Analysis

The results of the chlorophyll fluorescence analysis were split into (a) the whole leaflet response before herbivory as a mean value of all the AOIs per treatment, (b) the whole leaflet response after herbivory as a mean value of all the AOIs per treatment, and (c) as the response in three zones—feeding spots, surrounding zones, and rest of the leaflet area.

First, we compared the untreated and treated control treatments to evaluate if they were significantly different or could be combined. This was tested with a two-way (Herbivory and Root treatment) repeated measures ANOVA. Comparing the two controls before herbivory as well as after herbivory allowed us to determine in which measurements we could combine the two control treatments. Next, we tested if the fungi-inoculated plants had a different photosynthetic response before and after herbivory at the whole leaflet level. We tested this for all the photosynthetic parameters measured using a two-way repeated measures ANOVA with Root Treatment and Herbivory as the two factors, with post hoc comparisons using Dunn–Šidák correction. Finally, we analyzed whether the photosynthetic response to herbivory was different between leaf zones—feeding spot, surrounding area, or rest of the leaflet area—using a two-way ANOVA of the effect of Area and Root treatment on all measurements. Variance of homogeneity was verified with Levene’s test and the normality using Shapiro–Wilk test. Significance was estimated at a level of *p* < 0.05. Data analysis and graphs were obtained using IBM SPSS Statistics for Windows version 28.0.

## Figures and Tables

**Table 1:** Definitions of the measured chlorophyll fluorescence parameters.

Parameter	Definition	Calculation
Φ*_PSII_*	Effective quantum yield of PSII photochemistry	(F*m*΄ − F*s*)/F*m*΄ [52]
Φ*_NPQ_*	Quantum yield of regulated non-photochemical energy loss in PSII	F*s*/F*m*΄ − F*s*/F*m* [52]
Φ*_NO_*	Quantum yield of nonregulated loss in PSII	F*s*/F*m* [52]
NPQ	Non-photochemical quenching reflecting the dissipation of excitation energy as heat	(F*m* − F*m*΄)/F*m*΄ [82]
ETR	Electron transport rate	Φ_PSII_ × PAR × c × abs, where PAR is the photosynthetically active radiation, c is 0.5, and abs is the total light absorption of the leaf taken as 0.84 [83]
q*_p_*	Photochemical quenching, representing the fraction of open PSII reaction centers	(F*m*΄ − F*s*)/(F*m*΄ − F*o*΄) [84]
EXC	Excess excitation energy	(F*v*/F*m* − Φ_PSII_)/F*v*/F*m* [85]

## Data Availability

The data presented in this study are available in this article.

## Supplementary Materials

Table S1: Comparison of controls, Table S2: Percentage pairwise differences between experimental treatments for Φ*_PSII_* and Φ*_NPQ_*, Table S3: Percentage pairwise differences between experimental treatments for Φ*_NO_*, Table S4: Percentage pairwise differences between experimental treatments for NPQ and ETR, Table S5: Percentage pairwise differences between experimental treatments for q*_P_* and EXC, Table S6: Statistical significance of pairwise differences between different leaflet zones for Φ*_PSII_* and Φ*_NPQ_* Table S7: Statistical significance of pairwise differences between different leaflet zones for Φ*_NO_*, Figure S1: Graphs showing percentage changes in NPQ and ETR between treatments, Figure S2: Graphs showing percentage changes in q*_P_* and EXC.
