# Supplementary material for: Root-Associated Entomopathogenic Fungi Modulate Their Host Plant’s Photosystem II Photochemistry and Response to Herbivorous Insects
Source: Molecules. 2021 Dec 29;27(1):207. doi: 10.3390/molecules27010207 (PMC8746981; doi:10.3390/molecules27010207)
Supplement: Supplementary file 1 [file molecules-27-00207-s001.zip › molecules-1496080-supplementary.pdf]

Article

Root-Associated Entomopathogenic Fungi Modulate their Host Plant's Photosystem II Photchemistry and Response to Herbivorous Insects

Julietta Moustaka *, Nicolai Vitt Meyling and Thure Pavlo Hauser *

Table S1. Comparison of the Untreated and Treated Controls, showing P values for pairwise comparisons with post hoc tests and Dunn-Šidák correction.

Symbol	Parameter	Untreated vs Treated Control	
		Before	After
**Φ_{PSII}**	Effective quantum yield of PSII photochemistry	0.697	1
**Φ_{NPQ}**	Quantum yield of regulated non photochemical energy loss in PSII	1	1
**Φ_{NO}**	Quantum yield of non-regulated loss in PSII	**0.001**	1
**NPQ**	Non-photochemical quenching reflecting the dissipation of excitation energy as heat	0.582	0.999
**qP**	Electron transport rate	**0.029**	0.225
**ETR**	Photochemical quenching, representing the fraction of open PSII reaction centers	0.703	1
**EXS**	Excess excitation energy	0.33	0.797

Table S2. Percentage pairwise differences between the experimental treatments for Φ_{NPQ} (upper right yellow quadrant) and Φ_{PSII} (lower left blue quadrant). Green and red colors indicate increases and decreases, respectively, bold numbers indicate significant differences at p <0.05. Untreated and Treated Controls were combined; see Table S1.

Φ_{NPQ} / Φ_{PSII}		Before			After		
		**Control**	***M. brunneum***	***B. bassiana***	**Control**	***M. brunneum***	***B. bassiana***
**Before**	**Control**		**12**	**9**	**18**		
	M. brunneum	**15**		2		**22**	
	B. bassiana	8	8				**19**
**After**	**Control**	**20**				5	**7**
	M. brunneum		**32**		7		**3**
	B. bassiana			**24**	5	2	

Table S3. Percentage pairwise differences between the experimental treatments for q_P (upper right yellow quadrant) and Φ_{NO} (lower left blue quadrant). Green and red colors indicate increases and decreases, respectively, bold numbers indicate significant differences at $p < 0.05$. Untreated and Treated Controls were combined for q_P, see Table S1.

**q_P / Φ_{NO}**		**Before**				**After**		
		**Untreated Control**	**Treated Control**	***M. brunneum***	***B. bassiana***	**Controls**	***M. brunneum***	***B. bassiana***
**Before**	**Untreated Control**		**15**	**22**	**18**	6.5		
	**Treated Control**	**9**		9	4	**25**		
	M. brunneum	**8**	1		5		**27**	
	B. bassiana	3	**6**	**5**				**22**
**After**	**Controls**	**8.6**	0.16				**7**	**7**
	M. brunneum			**5**		4		0.27
	B. bassiana				3.7	**2**	**6**	

Table S4. Percentage pairwise differences between the experimental treatments for ETR (upper right yellow quadrant) and NPQ (lower left blue quadrant). Green and red colors indicate increases and decreases, respectively, bold numbers indicate significant differences at $p < 0.05$. Untreated and Treated Controls were combined, see Table S1.

ETR / NPQ		Before			After		
		**Control**	***M. brunneum***	***B. bassiana***	**Control**	***M. brunneum***	***B. bassiana***
**Before**	**Control**		**15**	8	**21**		
	M. brunneum	**9**		8		**32**	
	B. bassiana	**11**	2				**30**
**After**	**Control**	**22**				6	5
	M. brunneum		**28**		0		2
	B. bassiana			**23**	**9**	**9**	

Table S5. Percentage pairwise differences between the experimental treatments for EXC. Green and red colours indicate increases and decreases, respectively, bold numbers indicate significant differences at $p < 0.05$. Untreated and Treated Controls were combined, see Table S1.

**Excess Excitation Energy**		**Before**			**After**		
		**Control**	***M. brunneum***	***B. bassiana***	**Control**	***M. brunneum***	***B. bassiana***
**Before**	**Control**						
	M. brunneum	**12**					
	B. bassiana	**8**	4				
**After**	**Control**	**16**					
	**M. brunneum**		**19**		7		
	B. bassiana			**16**	7	0.03	

Table S6. Statistical significance of pairwise differences between different leaflet zones (feeding spot, surrounding zone, and rest of leaf) for Φ_{NPQ} (upper right yellow quadrant) and Φ_{NPQ} (lower left blue quadrant). Significance at *p <0.001* is indicated by ***, otherwise actual *p* values are given with significance at *p <0.05* indicated by bold type.

Φ_{NPQ} / Φ_{PSII}		**Control**			***M. brunneum***			***B. bassiana***		
		Feed	Surround	Rest	Feed	Surround	Rest	Feed	Surround	Rest
**Control**	Feed		***	***	0.17			***		
	Surround	***		0.96		0.65			0.11	
	Rest	***	1				0.18			0.13
M. brunneum	Feed	0.19				***	***	**0.03**		
	Surround		0.27		***		0.55		0.26	
	Rest			**0.04**	***	0.97				0.84
B. bassiana	Feed	**0.01**			0.37				***	***
	Surround		0.23			0.94		***		1
	Rest			0.18			0.42	***	0.99	

Table S7. Statistical significance of pairwise differences between different leaflet zones (feeding spot, surrounding zone, and rest of leaf) for Φ_{NO}. Significance at *p <0.001* is indicated by ***, at *p <0.01* by **, otherwise actual *p* values are given with significances at *p <0.05* indicated by bold type.

**ΦNO**		**Control**			***M. brunneum***			***B. bassiana***		
		Feed	Surround	Rest	Feed	Surround	Rest	Feed	Surround	Rest
**Control**	Feed									
	Surround	***								
	Rest	***	0.81							
M. brunneum	Feed	***								
	Surround		0.30		***					
	Rest			0.23	***	0.68				
B. bassiana	Feed	***			**					
	Surround		0.71			0.26		***		
	Rest			0.85			0.33	***	0.99	

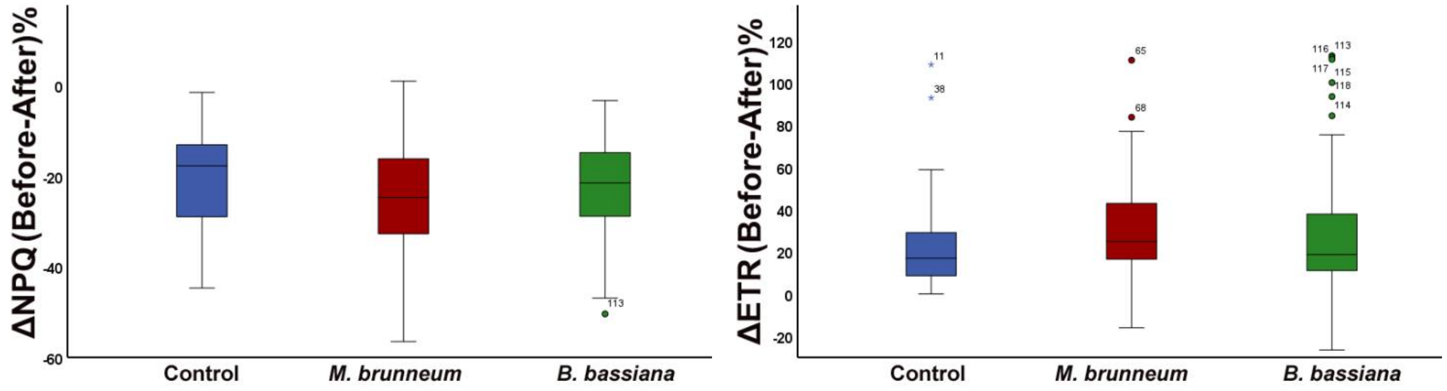

(**a**) (**b**)

Figure S1. Graphs showing percentage changes from Before to After herbivory for **(a)** non-photochemical quenching (NPQ) and **(b)** electron transport rate (ETR), shown for Control plants (blue), *M. brunneum* inoculated plants (red), and *B. bassiana* inoculated plants (green). Boxes and whiskers indicate the 10, 25, 50, 75 and 90 percentiles. Circles and asterisks indicate outliers.

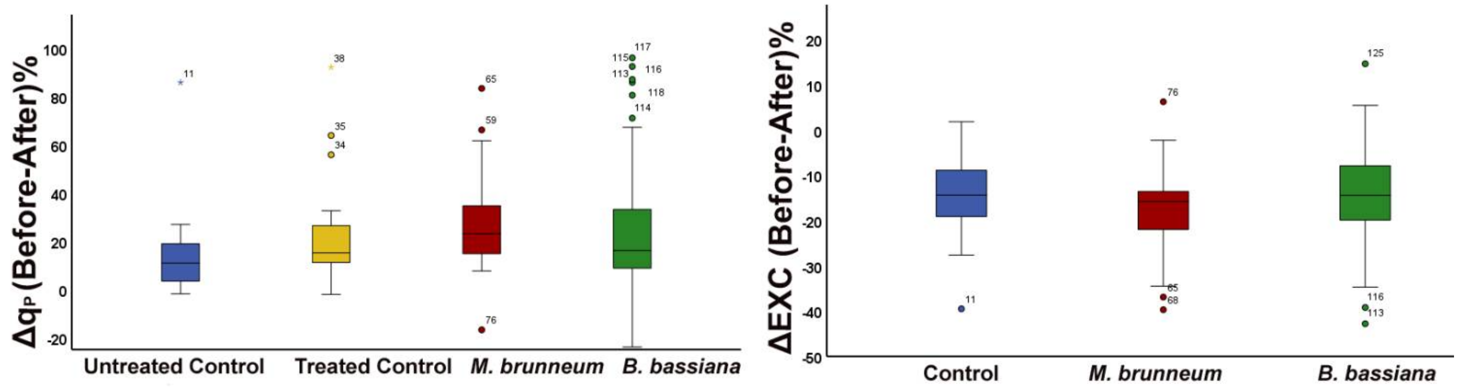

(**a**) (**b**)

Figure S2. Graphs showing percentage changes from Before to After herbivory for **(a)** photochemical quenching (q_P) and **(b)** excess excitation energy in PSII (EXC) , shown for Untreated and Treated Control plants (blue and yellow), *M. brunneum* inoculated plants (red), and *B. bassiana* inoculated plants (green). Boxes and whiskers indicate the 10, 25, 50, 75 and 90 percentiles. Circles and asterisks indicate outliers.
